# Supplementary material for: HIVprotI: an integrated web based platform for prediction and design of HIV proteins inhibitors
Source: J Cheminform. 2018 Mar 9;10:12. doi: 10.1186/s13321-018-0266-y (PMC5845081; doi:10.1186/s13321-018-0266-y)
Supplement: Supplementary file 2 — Additional file 2. Source code of HIVProtI web server. [file 13321_2018_266_MOESM2_ESM.zip › HIVprotI_Source-code/hivprotI/weka-3-6-6/doc/weka/datagenerators/classifiers/classification/BayesNet.html]

BayesNet


---


|  |  |  |
| --- | --- | --- |
| **Overview** \| **Package** \| **Class** \| **Tree** \| **Deprecated** \| **Index** \| **Help** \| **Weka's home** | |  |
| **PREV CLASS**   **NEXT CLASS** | **FRAMES**    **NO FRAMES**     **All Classes** |  |
| SUMMARY: NESTED \| FIELD \| CONSTR \| METHOD | DETAIL: FIELD \| CONSTR \| METHOD |  |


---


## weka.datagenerators.classifiers.classification Class BayesNet

```
java.lang.Object
  weka.datagenerators.DataGenerator
      weka.datagenerators.ClassificationGenerator
          weka.datagenerators.classifiers.classification.BayesNet
```

**All Implemented Interfaces:**: java.io.Serializable, OptionHandler, Randomizable, RevisionHandler

---

``` public class BayesNet extends ClassificationGenerator ```

Generates random instances based on a Bayes network.


Valid options are:

```
 -h
  Prints this help.
```

```
 -o <file>
  The name of the output file, otherwise the generated data is
  printed to stdout.
```

```
 -r <name>
  The name of the relation.
```

```
 -d
  Whether to print debug informations.
```

```
 -S
  The seed for random function (default 1)
```

```
 -n <num>
  The number of examples to generate (default 100)
```

```
 -A <num>
  The number of arcs to use. (default 20)
```

```
 -C <num>
  The cardinality of the attributes and the class. (default 2)
```

**Version:**
:   $Revision: 1.4 $

**Author:**
:   FracPete (fracpete at waikato dot ac dot nz)

**See Also:**: `BayesNetGenerator`, Serialized Form

---

**Constructor Summary**

`BayesNet()`
  
          initializes the generator


| **Method Summary** | |
| --- | --- |
| `java.lang.String` | `cardinalityTipText()`             Returns the tip text for this property |
| `Instances` | `defineDataFormat()`             Initializes the format for the dataset produced. |
| `Instance` | `generateExample()`             Generates one example of the dataset. |
| `Instances` | `generateExamples()`             Generates all examples of the dataset. |
| `java.lang.String` | `generateFinished()`             Generates a comment string that documentats the data generator. |
| `java.lang.String` | `generateStart()`             Generates a comment string that documentates the data generator. |
| `int` | `getCardinality()`             Gets the cardinality of the attributes (incl class attribute) |
| `int` | `getNumArcs()`             Gets the number of arcs for the bayesian net |
| `int` | `getNumAttributes()`             Gets the number of attributes that should be produced. |
| `int` | `getNumExamples()`             Gets the number of examples, given by option. |
| `java.lang.String[]` | `getOptions()`             Gets the current settings of the datagenerator. |
| `java.lang.String` | `getRevision()`             Returns the revision string. |
| `boolean` | `getSingleModeFlag()`             Return if single mode is set for the given data generator mode depends on option setting and or generator type. |
| `java.lang.String` | `globalInfo()`             Returns a string describing this data generator. |
| `java.util.Enumeration` | `listOptions()`             Returns an enumeration describing the available options. |
| `static void` | `main(java.lang.String[] args)`             Main method for executing this class. |
| `java.lang.String` | `numArcsTipText()`             Returns the tip text for this property |
| `java.lang.String` | `numAttributesTipText()`             Returns the tip text for this property |
| `void` | `setCardinality(int value)`             Sets the cardinality of the attributes (incl class attribute) |
| `void` | `setNumArcs(int value)`             Sets the number of arcs for the bayesian net |
| `void` | `setNumAttributes(int numAttributes)`             Sets the number of attributes the dataset should have. |
| `void` | `setNumExamples(int numExamples)`             Sets the number of examples, given by option. |
| `void` | `setOptions(java.lang.String[] options)`             Parses a list of options for this object. |

**Methods inherited from class weka.datagenerators.ClassificationGenerator**

`numExamplesTipText`

**Methods inherited from class weka.datagenerators.DataGenerator**

`debugTipText, defaultOutput, formatTipText, getDatasetFormat, getDebug, getNumExamplesAct, getOutput, getRandom, getRelationName, getSeed, makeData, outputTipText, randomTipText, relationNameTipText, seedTipText, setDatasetFormat, setDebug, setOutput, setRandom, setRelationName, setSeed`

**Methods inherited from class java.lang.Object**

`equals, getClass, hashCode, notify, notifyAll, toString, wait, wait, wait`

**Constructor Detail**

### BayesNet

```
public BayesNet()
```

:   initializes the generator


**Method Detail**

### globalInfo

```
public java.lang.String globalInfo()
```

:   Returns a string describing this data generator.

    :   **Returns:**: a description of the data generator suitable for displaying in the explorer/experimenter gui

---


### listOptions

```
public java.util.Enumeration listOptions()
```

:   Returns an enumeration describing the available options.

    :   **Specified by:**: `listOptions` in interface `OptionHandler` **Overrides:**: `listOptions` in class `ClassificationGenerator`
    :   **Returns:**: an enumeration of all the available options

---


### setOptions

```
public void setOptions(java.lang.String[] options)
                throws java.lang.Exception
```

:   Parses a list of options for this object.


    Valid options are:

    ```
     -h
      Prints this help.
    ```

    ```
     -o <file>
      The name of the output file, otherwise the generated data is
      printed to stdout.
    ```

    ```
     -r <name>
      The name of the relation.
    ```

    ```
     -d
      Whether to print debug informations.
    ```

    ```
     -S
      The seed for random function (default 1)
    ```

    ```
     -n <num>
      The number of examples to generate (default 100)
    ```

    ```
     -A <num>
      The number of arcs to use. (default 20)
    ```

    ```
     -C <num>
      The cardinality of the attributes and the class. (default 2)
    ```

    :   **Specified by:**: `setOptions` in interface `OptionHandler` **Overrides:**: `setOptions` in class `ClassificationGenerator`
    :   **Parameters:**: `options` - the list of options as an array of strings **Throws:**: `java.lang.Exception` - if an option is not supported

---


### getOptions

```
public java.lang.String[] getOptions()
```

:   Gets the current settings of the datagenerator.

    :   **Specified by:**: `getOptions` in interface `OptionHandler` **Overrides:**: `getOptions` in class `ClassificationGenerator`
    :   **Returns:**: an array of strings suitable for passing to setOptions **See Also:**: `DataGenerator.removeBlacklist(String[])`

---


### setNumAttributes

```
public void setNumAttributes(int numAttributes)
```

:   Sets the number of attributes the dataset should have.

    :   **Parameters:**: `numAttributes` - the new number of attributes

---


### getNumAttributes

```
public int getNumAttributes()
```

:   Gets the number of attributes that should be produced.

    :   **Returns:**: the number of attributes that should be produced

---


### numAttributesTipText

```
public java.lang.String numAttributesTipText()
```

:   Returns the tip text for this property

    :   **Returns:**: tip text for this property suitable for displaying in the explorer/experimenter gui

---


### setCardinality

```
public void setCardinality(int value)
```

:   Sets the cardinality of the attributes (incl class attribute)

    :   **Parameters:**: `value` - the cardinality

---


### getCardinality

```
public int getCardinality()
```

:   Gets the cardinality of the attributes (incl class attribute)

    :   **Returns:**: the cardinality of the attributes

---


### cardinalityTipText

```
public java.lang.String cardinalityTipText()
```

:   Returns the tip text for this property

    :   **Returns:**: tip text for this property suitable for displaying in the explorer/experimenter gui

---


### setNumArcs

```
public void setNumArcs(int value)
```

:   Sets the number of arcs for the bayesian net

    :   **Parameters:**: `value` - the number of arcs

---


### getNumArcs

```
public int getNumArcs()
```

:   Gets the number of arcs for the bayesian net

    :   **Returns:**: the number of arcs

---


### numArcsTipText

```
public java.lang.String numArcsTipText()
```

:   Returns the tip text for this property

    :   **Returns:**: tip text for this property suitable for displaying in the explorer/experimenter gui

---


### setNumExamples

```
public void setNumExamples(int numExamples)
```

:   Sets the number of examples, given by option.

    :   **Overrides:**: `setNumExamples` in class `ClassificationGenerator`
    :   **Parameters:**: `numExamples` - the new number of examples

---


### getNumExamples

```
public int getNumExamples()
```

:   Gets the number of examples, given by option.

    :   **Overrides:**: `getNumExamples` in class `ClassificationGenerator`
    :   **Returns:**: the number of examples, given by option

---


### getSingleModeFlag

```
public boolean getSingleModeFlag()
                          throws java.lang.Exception
```

:   Return if single mode is set for the given data generator
    mode depends on option setting and or generator type.

    :   **Specified by:**: `getSingleModeFlag` in class `DataGenerator`
    :   **Returns:**: single mode flag **Throws:**: `java.lang.Exception` - if mode is not set yet

---


### defineDataFormat

```
public Instances defineDataFormat()
                           throws java.lang.Exception
```

:   Initializes the format for the dataset produced.
    Must be called before the generateExample or generateExamples
    methods are used.
    Re-initializes the random number generator with the given seed.

    :   **Overrides:**: `defineDataFormat` in class `DataGenerator`
    :   **Returns:**: the format for the dataset **Throws:**: `java.lang.Exception` - if the generating of the format failed **See Also:**: `DataGenerator.getSeed()`

---


### generateExample

```
public Instance generateExample()
                         throws java.lang.Exception
```

:   Generates one example of the dataset.

    :   **Specified by:**: `generateExample` in class `DataGenerator`
    :   **Returns:**: the generated example **Throws:**: `java.lang.Exception` - if the format of the dataset is not yet defined: `java.lang.Exception` - if the generator only works with generateExamples which means in non single mode

---


### generateExamples

```
public Instances generateExamples()
                           throws java.lang.Exception
```

:   Generates all examples of the dataset. Re-initializes the random number
    generator with the given seed, before generating instances.

    :   **Specified by:**: `generateExamples` in class `DataGenerator`
    :   **Returns:**: the generated dataset **Throws:**: `java.lang.Exception` - if the format of the dataset is not yet defined: `java.lang.Exception` - if the generator only works with generateExample, which means in single mode **See Also:**: `DataGenerator.getSeed()`

---


### generateStart

```
public java.lang.String generateStart()
```

:   Generates a comment string that documentates the data generator.
    By default this string is added at the beginning of the produced output
    as ARFF file type, next after the options.

    :   **Specified by:**: `generateStart` in class `DataGenerator`
    :   **Returns:**: string contains info about the generated rules

---


### generateFinished

```
public java.lang.String generateFinished()
                                  throws java.lang.Exception
```

:   Generates a comment string that documentats the data generator.
    By default this string is added at the end of theproduces output
    as ARFF file type.

    :   **Specified by:**: `generateFinished` in class `DataGenerator`
    :   **Returns:**: string contains info about the generated rules **Throws:**: `java.lang.Exception` - if the generating of the documentaion fails

---


### getRevision

```
public java.lang.String getRevision()
```

:   Returns the revision string.

    :   **Returns:**: the revision

---


### main

```
public static void main(java.lang.String[] args)
```

:   Main method for executing this class.

    :   **Parameters:**: `args` - should contain arguments for the data producer:


---


|  |  |  |
| --- | --- | --- |
| **Overview** \| **Package** \| **Class** \| **Tree** \| **Deprecated** \| **Index** \| **Help** \| **Weka's home** | |  |
| **PREV CLASS**   **NEXT CLASS** | **FRAMES**    **NO FRAMES**     **All Classes** |  |
| SUMMARY: NESTED \| FIELD \| CONSTR \| METHOD | DETAIL: FIELD \| CONSTR \| METHOD |  |


---
